# Gene Expression of Type VI Secretion System Associated with Environmental Survival in *Acidovorax avenae* subsp. *avenae* by Principle Component Analysis

**DOI:** 10.3390/ijms160922008

**Published:** 2015-09-11

**Authors:** Zhouqi Cui, Guoqiang Jin, Bin Li, Kaleem Ullah Kakar, Mohammad Reza Ojaghian, Yangli Wang, Guanlin Xie, Guochang Sun

**Affiliations:** 1 State Key Laboratory of Rice Biology, Institute of Biotechnology, Zhejiang University, Hangzhou 310058, China; E-Mails: twelue@163.com (Z.C.); k_khan_2009@yahoo.com (K.U.K.); smro59@gmail.com (M.R.O.); glxie@zju.edu.cn (G.X.); 2 Yuhang Extension and Service Center of Agriculture Technical, Hangzhou 311100, China; E-Mail: wzli@126.com; 3 State Key Laboratory Breeding Base for Zhejiang Sustainable Plant Pest and Disease Control, Key Laboratory of Detection for Pesticide Residues, Ministry of Agriculture, Zhejiang Academy of Agricultural Sciences, Hangzhou 310021, China; E-Mail: ylwang88@aliyun.com

**Keywords:** T6SS gene expression, gene knockout, co-culture, *in vivo*, stress, principle component analysis

## Abstract

Valine glycine repeat G (VgrG) proteins are regarded as one of two effectors of Type VI secretion system (T6SS) which is a complex multi-component secretion system. In this study, potential biological roles of T6SS structural and VgrG genes in a rice bacterial pathogen, *Acidovorax avenae* subsp. *avenae* (*Aaa*) RS-1, were evaluated under seven stress conditions using principle component analysis of gene expression. The results showed that growth of the pathogen was reduced by H_2_O_2_ and paraquat-induced oxidative stress, high salt, low temperature, and *vgrG* mutation, compared to the control. However, pathogen growth was unaffected by co-culture with a rice rhizobacterium *Burkholderia seminalis* R456. In addition, expression of 14 T6SS structural and eight *vgrG* genes was significantly changed under seven conditions. Among different stress conditions, high salt, and low temperature showed a higher effect on the expression of T6SS gene compared with host infection and other environmental conditions. As a first report, this study revealed an association of T6SS gene expression of the pathogen with the host infection, gene mutation, and some common environmental stresses. The results of this research can increase understanding of the biological function of T6SS in this economically-important pathogen of rice.

## 1. Introduction

Secretion of proteins through secretion systems is a way by which bacteria influence their extracellular surroundings and other bacteria [1]. In pathogenic bacteria, the secretion systems which transfer proteins and toxins into the environment and within a eukaryotic target cell are important for their virulence and survival in hosts [2,3]. At least six distinct multi-component secretion systems (referred to type I–VI secretion system, or T1SS–T6SS) are used by Gram-negative bacterial pathogens to transport the proteins across the membranes of the bacteria and, eventually, the host [4,5,6,7,8]. The T6SS is a newly found multi-component secretion system, which is often involved in interaction with eukaryotic hosts in either pathogenic or symbiotic relationships [9]. According to previous studies, most of T6SS-containing bacteria are known as human and animal pathogens [7,10]. The T6SS encoded by clusters of contiguous genes is composed of 13 conserved proteins and a variable complement of accessory elements. It is reported that they are present in one or more copies in numerous Gram-negative bacterial pathogens including *Vibrio cholerae*, *Pseudomonas aeruginosa*, *Yersinia pestis*, *Escherichia coli*, *Salmonella enterica*, *Agrobacterium tumefaciens*, *Rhizobium leguminosarum*, *Francisellatularensis*, *Burkholderia mallei*, and *Edwardsiella* spp. In addition, the T6SS genes have key roles in virulence-related processes in some of these bacterial pathogens [11,12,13,14,15,16,17,18].

In bacterial pathogens, infection of the host depends on effective colonization, as well as survival, of the pathogen in the host and environment by resistance against different stress conditions [19,20,21]. Researchers have shown that T6SS plays an important role in pathogens to show resistance to environmental stresses [22,23,24]. For example, *pppA-ppkA* null mutant decreased resistance to H_2_O_2_-induced oxidative stress of *P. aeruginosa* [25]. Weber *et al.* (2009) reported a new role for T6SS in the ecology of *Vibrio anguillarum* and attributed this role to a signal-sensing mechanism that modulates expression of regulators of the general stress response [26]. These results indicate that there is a potential interaction between stress conditions and T6SS genes in bacterial pathogens of human and animals.

Recently, T6SSs of plant pathogens such as *Pseudomonas syringae* and *Pectobacterium wasabiae* have been studied [27,28,29]. Furthermore, genome-wide analysis revealed the existence of only one T6SS gene cluster in strain RS-1 of *Acidovorax avenae* subsp. *avenae* (*Aaa*), a widely-distributed seed-borne pathogen of rice [30]. In addition, our previous study showed that the homolog of VgrG, as one of two most important putative T6SS effectors, cannot be encoded by a T6SS cluster but it is considered as an orphan component in *Aaa* RS-1 [31]. The objective of this study was to assess the potential interaction between expression of T6SS structure and VgrG genes of *Aaa* RS-1 and common environmental stress conditions, including *in vivo* infection, co-culture with rice rhizobacterium *Burkholderia seminalis* R456, and one *vgrG* (Aave_0497) mutation, as well as high salt, low-temperature, H_2_O_2_- and paraquat-induced stresses.

## 2. Results

### 2.1. Repression of Bacterial Growth under Environmental Stress Conditions

**Figure 1:**
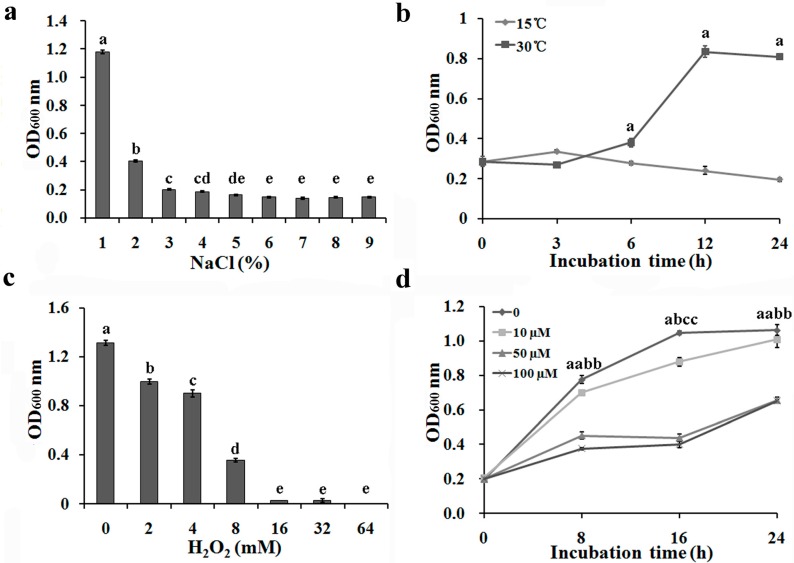
Growth of *Acidovorax avenae* subsp. *avenae* RS-1 under different conditions of (**a**) NaCl-induced osmotic stress; (**b**) low temperature stress; (**c**) H_2_O_2_-mediated oxidative stress; (**d**) paraquat-mediated oxidative stress (*p* < 0.05). *Aaa* RS-1 incubated in NA broth with 1.0% NaCl at 30 °C, 200 rpm for 24 h was used as the negative control. Data from the repeated experiment were pooled and subjected to analysis of variance. Columns with the same letters (a–e) are not significantly different (*p* = 0.05). Error bars represent the standard error of the mean.

The growth of *Aaa* strain RS-1 was significantly inhibited by high salt, low temperature, H_2_O_2_- and paraquat-mediated oxidative stress compared to the corresponding control. The survival of *Aaa* RS-1 under conditions of high salt, low temperature, H_2_O_2_ and paraquat stress is shown in Figure 1. Results from this study indicated that nutrient broth (NB) supplemented with 2.0% and 3.0% NaCl caused a reduction for 66.1% and 83.1%, respectively, in the growth of *Aaa* RS-1 compared to the control (1.0% NaCl). No statistical difference was observed among the concentrations of NaCl higher than 3.0% (Figure 1a). Compared to 30 °C (control), low temperature at 15 °C caused a 27.1%, 71.2%, and 75.9% reduction in the growth of *Aaa* RS-1 after 6, 12, and 24 h, respectively (Figure 1b). Furthermore, H_2_O_2_ at 8, 16, and 32 mM caused a significant reduction of 73.0%, 98.2%, and 98.2%, respectively, in the growth of *Aaa* RS-1 (Figure 1c). Paraquat at 10, 50, and 100 µM caused a 16.0%, 58.0%, and 61.8% reduction, respectively, in the growth of *Aaa* RS-1 after 24 h compared to the control. The growth of *Aaa* RS-1 was unaffected by 10 µM of paraquat, but was significantly reduced by 50 and 100 µM of paraquat (Figure 1d). In addition, NB supplemented with 2.0% NaCl, culture at 15 °C, NB supplemented with 8 mM H_2_O_2_ and NB supplemented with 50 µM paraquat for 24 h were selected as repression points for salt-induced osmotic stress, low temperature stress, H_2_O_2_-induced oxidized stress, and paraquat-induced oxidized stress, respectively.

### 2.2. Repression of Bacterial Growth during in Vivo Infection and Co-Culture Condition

We did not count the number of bacteria during *in vivo* infection and did not compare with the number of bacteria cultured under *in vitro* condition because the bacterial density is obviously higher in the broth *in vitro* than in the plants *in vivo*. Furthermore, the growth of *Aaa* strain RS-1 was unaffected by the co-culture of bacteria with *B. seminalis* R456. The OD_600_ value increased from 0.2 to 1.4 when bacteria were incubated in NB alone, while the OD_600_ value increased from 0.2 to 1.1 after 24 h at 30 °C when bacteria were co-cultured in NB with *B. seminalis* R456.

### 2.3. Repression of Bacterial Growth in ΔvgrG-2

**Figure 2:**
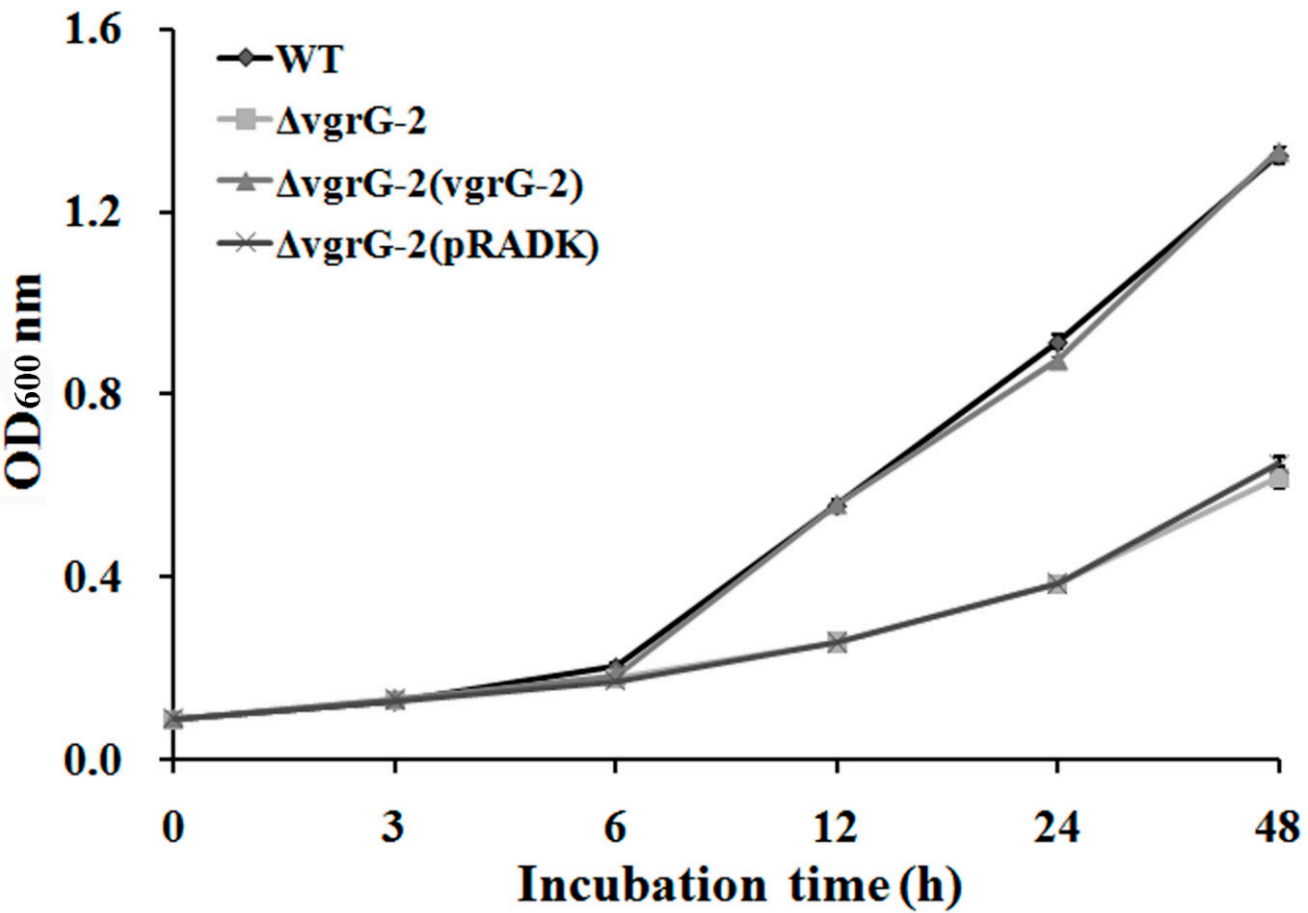
Comparison of growth between the wild type and Δ*vgrG*-2 mutant of *Acidovorax avenae* subsp. *avenae* strain RS-1. WT: wild type strain; Δ*vgrG*-2: *vgrG*-2 mutant strain; Δ*vgrG*-2(vgrG-2): *vgrG*-2 complementary strain; Δ*vgrG*-2(pRADK): mock strain with empty pRADK plasmid. Data from the repeated experiment were pooled and subjected to analysis of variance. Error bars represent the standard error of the mean.

There was a significant difference in the growth between the wild type and the Δ*vgrG*-2 of *Aaa* strain RS-1. The OD_600_ of wild type increased from 0.089 to 0.545, 0.910, and 1.348 after 12, 24, and 48 h of incubation at 30 °C, respectively. However, the OD_600_ of Δ*vgrG*-2 was significantly inhibited by 50.6%, 57.1%, and 52.7% compared to the wild type after 12, 24, and 48 h of incubation at 30 °C, respectively. There was no significant difference in the growth between the wild type and complementary strain Δ*vgrG*-2(vgrG-2). In addition, there was no significant difference in the growth between the mutant strain and mock strain Δ*vgrG*-2(pRADK) (Figure 2).

### 2.4. No Significant Difference for in Vitro Expression of T6SS Gene

**Figure 3:**
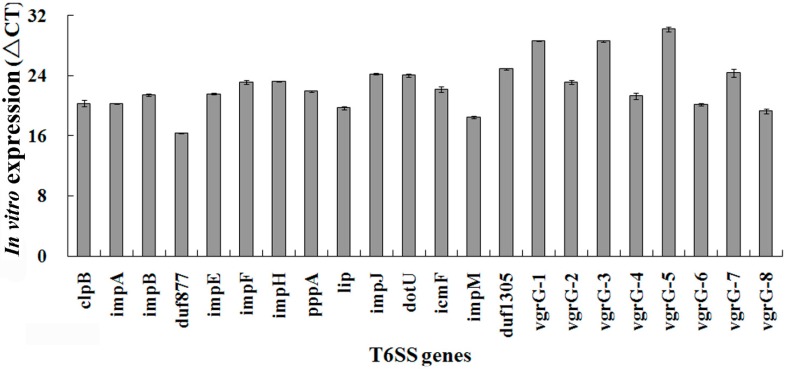
T6SS gene expression compared to 16S RNA gene, using quantitative real-time PCR in *Acidovorax avenae* subsp. *avenae* RS-1 under *in vitro* condition.

Gene expression data obtained from quantitative real-time PCR (qPCR) showed that the Δ*C*_t_ values of 22 T6SS genes (14 structural genes and eight *vgrG* genes) in NB (control) samples varied from 16.4 to 30.2 (Δ*C*_t_) (Figure 3). This suggested that there was a slight difference in the gene expression of each T6SS component under *in vitro* condition. However, in general, this result revealed that the expression level of 22 different T6SS genes under *in vitro* condition was similar for this change is less than two-fold, which have been regarded as the standard to differentiate significant change from insignificant change.

### 2.5. Different Expression of T6SS Gene under Various Environmental Conditions

Differential changes were observed in T6SS gene expression of *Aaa* strain RS-1 during *in vivo* rice infection, co-culture with *B. seminalis* R456, and *vgrG*-2 mutation as well as high salt, low-temperature, H_2_O_2_- and paraquat-mediated oxidative stress. The expression of 22 T6SS genes was dependent on both the kind of gene and the type of condition (Table 1). Compared to the corresponding control, it was obvious that salt stress and low temperature up-regulated expression of most genes. On the contrary, the expression of most genes was down-regulated during *in vivo* infection and in H_2_O_2_- and paraquat-induced oxidized stress. In addition, none of the 22 T6SS genes was up-regulated in the *vgrG*-2 mutant.

**Table 1:** Gene expression of T6SS using quantitative real-time PCR in *Acidovorax avenae* subsp. *avenae* RS-1 during *in vivo* rice infection, *vgrG-*2 mutant, and co-culture with rice rhizobacterium *Burkholderia seminalis* R456, as well as high salt, low temperature, H_2_O_2_- and paraquat-mediated oxidative stress.

T6SS Gene	Expression Change Relative to that of *in Vitro* under the Conditions of						
	Salt ^a^	Temperature	Paraquat	H_2_O_2_	Co-Culture	*in Vivo*	Mutant
*clpB*	↑1.3	↑2.8 *	↓14.8 *	↓4.5 *	↑15.0 *	↓4.4 *	↓1140.8 *
*impA*	↓12.3 *	↑36.1 *	↓5.1 *	↓1.8	↓1.8	↓3.1 *	↓14.1 *
*impB*	↑20.9 *	↑2.6 *	↓1.1	↑4.4 *	↑7.4 *	↑2.6 *	↓27.0 *
*duf877*	↓40.1 *	↓1.5	↓7.9 *	↓10.2 *	↑1.6	↓2.2 *	↓3008.4 *
*impE*	↑2.0 *	↑3.8 *	↓4.5 *	↓2.0 *	↑1.4	↑1.9	↓28.8 *
*impF*	↑23.2 *	↑63.5 *	↑9.5 *	↑1.5	↑3.4 *	↑4.2 *	↓11.8 *
*impH*	↑3.8 *	↑1.2	↓1.0	↓2.7 *	↓1.0	↓1.3	↓4.3 *
*pppA*	↑5.1 *	↑2.7 *	↓8.3 *	↓1.7	↑4.2 *	↑1.8	↓17.7 *
*lip*	↑5.7 *	↓2.0 *	↓5.9 *	↓11.9 *	↓5.7 *	↓5.7 *	↓31.6 *
*impJ*	↑51.2 *	↑79.0 *	↓1.6	↑2.9 *	↑5.0 *	↓1.4	↓15.1 *
*dotU*	↓6.4 *	↓16.9 *	↓15.4 *	↓37.6 *	↓1.7	↓10.9 *	↓10.4 *
*icmF*	↑13.6 *	↑2.4 *	↑1.4	↑1.1	↑2.0 *	↓1.9	↓35.8 *
*impM*	↑2.4 *	↑2.5 *	↓6.2 *	↓2.2 *	↓2.7 *	↓1.5	↓400.6 *
*duf1305*	↑39.5 *	↑115.5 *	↓1.1	↑2.0 *	↑8.2 *	↑2.5 *	↓3.4 *
*vgrG-1*	↑1.0	↓1.2	↓1.4	↓2.2 *	↑1.7	↑1.0	↓1.8
*vgrG-2*	↑1.1	↑1.7	↓1.8	↑1.1	↑2.4 *	↑2.4 *	↓2.1 *
*vgrG-3*	↓4.0 *	↑1.6	↑3.0 *	↓4.4 *	↓2.1 *	↓3.8 *	↓2.5 *
*vgrG-4*	↑4.8 *	↑17.1 *	↑3.1 *	↑1.7	↑1.9	↑1.7	↓66.1 *
*vgrG-5*	↑20.6 *	↑597.0 *	↑1028.9 *	↑77.4 *	↑20.1 *	↑108.3 *	↑123.9 *
*vgrG-6*	↑1.1	↓1.8	↓14.5 *	↓7.0 *	↓6.5 *	↓2.2 *	↓16.8 *
*vgrG-7*	↑1.6	↓1.4	↑2.8 *	↓1.5	↓3.6 *	↑1.1	↑1.1
*vgrG-8*	↓1.9	↓4.0 *	↓18.1 *	↓9.4 *	↓6.6 *	↓3.5 *	↓11.1 *

^a^ Salt: 2% NaCl high salt; Temperature: 15 °C low temperature; Co-culture: co-culture with rice rhizobacterium *Burkholderia seminalis* R456; Mutant: *vgrG-*2 gene knockout mutant; H_2_O_2_: 8 mM H_2_O_2_-mediated oxidative stress; Paraquat: 50 µM paraquat-mediated oxidative stress; *In vivo*: *in vivo* rice infection. ↑: up-regulation; ↓: down-regulation; *: the change of gene expression is more than two fold compared to the corresponding *in vitro* control.

In this study, the transcript of almost all structural genes (except *dotU*) and three *vgrG*s (*vgrG-*2, *vgrG-*4, *vgrG*-6) showed their maximum repression level of expression in response to*vgrG*-2 mutant. Five structural genes (*impB*, *impH*, *pppA*, *liP* and *imcF*) showed their maximum activation level of expression in response to in salt stress; six structural genes (*impA*, *impE*, *impF*, *impJ*, *impM*, *duf1305*) and one *vgrG*-4 showed their maximum activation level of expression under low temperature stress. Based on the results, only one structural gene *clpB* and one *vgrG*-2 showed the highest level of expression under co-culture condition; three *vgrG*s (*vgrG-*3, *vgrG-*5, *vgrG*-7) showed their maximum activation level of expression under paraquat stress; and two *vgrG*s (*vgrG-*1 and *vgrG-*3) showed their maximum repression level of expression under H_2_O_2_ stress. Interestingly, no gene showed its special expression levels during *in vivo* infection (Table 1).

Salt stress caused differential expression of 16 genes (13 structural genes and three *vgrG*s), including 12 up-regulated genes and four down-regulated genes. Low temperature caused differential expression of 15 genes (12 structural genes and three *vgrG*s), including 12 up-regulated genes and three down-regulated genes. H_2_O_2_ stress caused differential expression of 15 genes (10 structural genes and five *vgrG*s), including four up-regulated genes and 11 down-regulated genes. In addition, Paraquat stress caused differential expression of 15 genes (nine structural genes and six *vgrG*s), including five up-regulated genes and 10 down-regulated genes. Co-culture caused differential expression of 15 genes (nine structural genes and six *vgrG*s), including nine up-regulated genes and six down-regulated genes. *In vivo* infection caused differential expression of 13 genes (eight structural genes and five *vgrG*s), including five up-regulated genes and eight down-regulated genes. None of the 14 structural genes was significantly up-regulated in the *vgrG*-2 mutant, but found to be down-regulated. 

### 2.6. Principle Component Analysis

**Figure 4:**
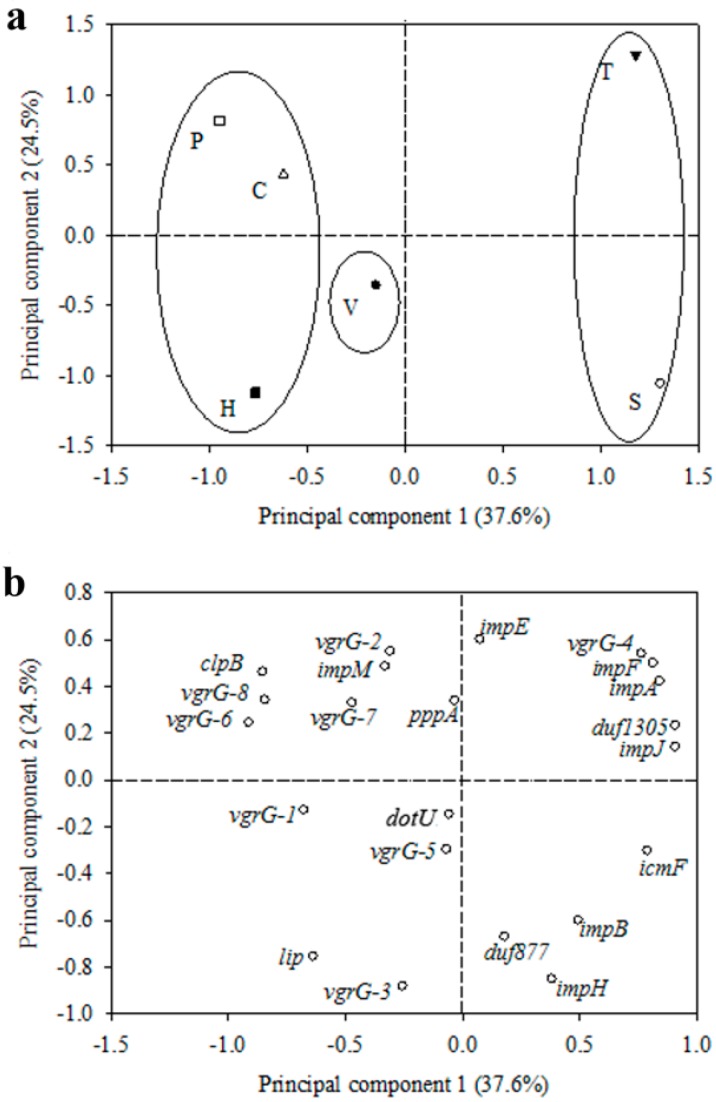
Two-dimensional principle component analyses (PCA) of 14 T6SS genes expression of *Acidovorax avenae* subsp. *avenae* RS-1 under 6 different conditions. (**a**) Scores from six different conditions; (**b**) loadings of the individual gene from the PCA of the relative expression data. S: 2% NaCl high salt; T: 15 °C low temperature; H: 8 mM H_2_O_2_-mediated oxidative stress; P: 50 µM paraquat-mediated oxidative stress; C: Co-culture with rice rhizobacterium *Burkholderia seminalis* R456; V: *In vivo* rice infection. The *vgrG*-2 mutation was excluded in PCA for the oversize change in several T6SS genes expressions. The different kinds of conditions were grouped into two groups and significantly separated from left to right along the PC1 axis in the order of (paraquat, H_2_O_2_, co-culture), (*in vivo*) and (temperature, salt) (*p* < 0.001).

The relative expression (RE) levels of 22 T6SS genes under six stress conditions (no *vgrG* mutant condition) were subjected to principle component analysis (PCA). The data for gene expression in mutant condition was excluded from analyses because of the oversize (incalculable) change in the expression levels of several genes. The first and second principal components of PCA plot (PC1 and PC2) accounted for 37.6%, and 24.5% of the variation, respectively, in the dataset (Figure 4). The contribution of conditions and genes as well as their interactions were determined based on the scores of PC1 and PC2. The relative importance of stress conditions and genes was determined based on their distance to the origin, the point where the two axes cross at zero on both scales. More distant (stress conditions and genes) from the origin was considered as more important factor for the expression levels.

The PCA results of condition factors in this study indicated that low temperature and high salt stresses were the most important contribution to the variation of RE levels, followed by H_2_O_2_, paraquat and co-culture stresses, while *in vivo* infection has the least contribution to the variation of RE levels based on their distance to the origin (Figure 4a). In addition, this study revealed three different types of contribution of stress types to the variation of RE levels. The different kinds of stress were grouped into three groups and significantly separated from left to right along the PC1 axis (*p* < 0.001) in order of group 1: (paraquat, H_2_O_2_, co-culture), group 2: (*in vivo*) and group 3: (temperature, salt).

Combining the PC1 axis and PC2 axis, the loadings of individual gene RE levels distributed on each quadrant indicated those genes are more related to these conditions that were on the same quadrant. The 22 T6SS genes were distributed in four quadrants by PC1 and PC2 axis. Some individual genes, like *pppA*, *dotU* and *vgrG-*5 were noted. *pppA* was close to PC2 axis, which indicated that *pppA* may be not only interact with paraquat and co-culture conditions but also with temperature condition. Furthermore, *dotU* and *vgrG-*5 were close to origin, revealing that they may be able to, at least partially, interact with almost all conditions (Figure 4b). However, *dotU* and *vgrG-*5 have the least contribution to the RE levels based on the distance to the origin.

## 3. Discussion

Bacterial pathogens are often exposed to various kinds of environmental stresses during host infection. Furthermore, a particular bacterial species is likely to encounter numerous taxonomically-different competitors in host and natural ecosystems [32]. Therefore, the molecular survival mechanism of bacteria is mainly dependent on their adaptation to the different hosts and environmental conditions. Identification of specific genes and gene expression patterns is important for studying the host infection and natural environmental survival of a bacterial pathogen. Recently, a review of T6SS indicated that T6SS may play key roles in microbial communities leading to more contributions to microbial interactions for environmental benefits [33]. Although T6SS is involved in a series of bacterial process, no research has been carried out to assess the effect of T6SS and its expression to host infection and environmental stresses. On the other hand, VgrGs, as one of the two putative effectors of T6SSs, are considered to play an important role in the function of T6SS machine [34,35,36,37]. Therefore, ecological roles of T6SS in *Aaa* RS-1 could be, at least in part, determined by understanding the effect of host infection, natural environmental stresses and gene mutation on the expression pattern of T6SS structural genes and *vgrG*s.

In agreement with previous research, this study revealed that bacteria adapt to different conditions such as low temperature, osmotic, and oxidative-induced stresses by changing the growth rate. It is known that bacteria are often subject to these stresses in the natural environment [19,38,39]. However, the extent of the changes depends on the type of conditions. T6SS was also found to be able to response to a variety of environmental conditions. In this study, four common environmental stress conditions were chosen to get the stress repression point for the growth of *Aaa* RS-1 during *in vivo* infection, co-culture with *B. seminalis* R456 (a rice rhizobacterium) and *vgrG*-2 mutation, to evaluate the efficacy of T6SS gene expression in adaptation to these seven stress conditions. Although a broad set of differentially-expressed genes have been observed in the response of bacteria to osmotic stress [25,40], no research was found about the role of T6SS in bacterial response to the salt stress. However, in this study, the result of PCA indicated that salt stress was one of the most important stress effectors interacted with T6SS gene expression. Four T6SS structure genes including *icmF*, *impB*, *impH*, and *duf877* were tightly interacted with salt-induced osmotic stress in PCA suggesting that the formation of T6SS structure may be related with the response of *Aaa* RS-1 to salt stress. Furthermore, little information is available about the role of VgrGs in bacterial response to environmental stresses. In this study, it became clear that the expression of most *vgrG*s (except *vgrG-*4) is affected by paraquat stress, H_2_O_2_ stress, and co*-*culture condition. However, the expression level of some *vgrG*s was not equal in paraquat stress and co*-*culture conditions, which suggest that multiple VgrGs in *Aaa* RS-1 maybe not be the result of a duplication but rather a gain of specific function, such as complementary expression in stress conditions. Although paraquat and H_2_O_2_ have a similar induced-oxidase mechanism, they were placed in the same group, but different spatial compartment, of PCA, indicating the complexity of the interaction between T6SS especially VgrGs with paraquat and H_2_O_2_.

T6SS has been experimentally shown to play a role in virulence in many cases. It may have an effect on limiting bacterial replication or virulence, increasing interaction with hosts other than pathogenesis which can lead to commensal or mutualistic conditions [41]. In some cases, putative T6SS components contribute to virulence, but in a manner that appears to be independent of other T6SS components [41]. Furthermore, expression of T6SS gene has been initially discovered as being specifically induced *in vivo* in many animal and human pathogens [42,43]. In this study, *in vivo* expressions of more than 50% of T6SS genes (13 of 22) were significantly changed, which showed that the T6SS genes were involved in the interaction between *Aaa* RS-1 and host. However, in contrast with the up-regulation in other animal and human pathogens [42,43], expression of most genes was down-regulated among 13 genes in *Aaa* RS-1, suggesting the complexity of T6SS expression in plant pathogenic bacteria. On the other hand, the transcriptomic level of gene expression may be not always having a positive correlation with translation level of protein. Several studies also revealed that T6SS, in most cases, are not critical factors of pathogenesis, but rather improve the efficiency of colonization and/or infection of bacterial pathogens by attacking or killing the other bacteria [44]. Similar to the result of previous studies [44], this study also revealed that the expression of 15 genes was altered in *Aaa* RS-1 when confronted with rice rhizospheric bacterium *B. Seminalis*. It may indicate that T6SSs can, at least in part, contribute to the interaction of *Aaa* RS-1 with other bacteria.

The result of PCA indicated that two genes including *dotU* and *vgrG-*5 were very close but near to the origin point. This suggests that the two genes may be able to interact with all conditions such as *in vivo* infection condition. Interestingly, the *in vivo* expression change of *dotU* (OmpA/MotB) was more than 10-fold compared to the control (*in vitro*), suggesting that *dotU* has stronger response to host infection than other T6SS genes. Furthermore, in agreement with Broms *et al.* (2012) [37], the role of *dotU* in the virulence of *Aaa* RS-1 was justified by the fact that its mutant lost or significantly reduced virulence to rice plants (data not shown). In addition, our previous study revealed that OmpA/MotB domain containing proteins was *in vivo* expressed in *Aaa* RS-1 [32], while the interaction between DotU and IcmF has also been identified in *Aaa* RS-1 (data not shown). In this study, there was an indirect correlation between the expressions level of *dotU* and *icmF* in all six conditions of qPCR, indicating the negative-interaction between them.

PCA revealed a slight effect of *vgrG-*5 as it was close to the origin point. However, the expression of *vgrG-*5 was significantly changed in all conditions. The contrast result may be due to this fact that qPCR revealed differences in expression of T6SS gene under the same condition or one T6SS gene under seven conditions. In addition to *vgrG-*5, study of the other VgrG-coding genes is a good area of research because they encode the most important putative T6SS effectors. In this study, eight *vgrG*s which have high homology to each other in *Aaa* RS-1 were picked up for examination of gene expression. The result showed that the *vgrG*s were more sensitive to the stress group of (paraquat, H_2_O_2_, co-culture) than the stress group of (temperature, salt).

This study revealed the considerable changes of T6SS gene expression in *Aaa* RS-1 under *in vivo* infection, co-culture with rhizobacterium, and *vgrG*-2 mutant as well as high salt, low-temperature, H_2_O_2_- and paraquat-induced oxidative stresses. The extent of the changes depended on the type of conditions. The expression of the T6SS gene under these stress conditions was analyzed by qPCR. As a first study, PCA was used in exploring the potential interaction between T6SS gene expression and stress conditions. In general, the result of qPCR and PCA showed that high salt and low temperature had a higher impact on expression of T6SS structural genes whereas expression of *vgrG*s was more sensitive to H_2_O_2_- and paraquat-induced oxidative stresses. Expression of almost all T6SS structural genes was highly repressed in *vgrG-2* mutant. Therefore, this study might provide a clue for further studies about the role of T6SS in the response of *Aaa* RS-1 to host infection, taxonomically different competitors, gene mutation, and various kinds of environmental stresses.

## 4. Experimental Section

### 4.1. Bacterial Strains, Plasmids and Chemicals

Bacteria and plasmids used in this study are listed in Table 2. *Aaa* RS-1 and *B. seminalis* R456 were isolated from diseased rice plants [45] and rice rhizosphere [46], respectively. Bacteria were stored in 20%–30% sterile glycerol (Shanglin Industries, Hangzhou, China) at −80 °C. Sodium chloride (NaCl) was obtained from Shisihewei Chemical Reagent Co., Ltd. (Shanghai, China), H_2_O_2_ was obtained from Sinopharm Chemical Reagent Co., Ltd. (Shanghai, China), and *paraquat* (*paraquat* dichloride X-hydrate) was purchased from Sigma-Aldrich (St. Louis, MO, USA). Bacterial strains were regularly cultured and maintained at initial concentration of 10^5^ CFU/mL in NB (peptone 10.0 g/mL, yeast extract 3.0 g/mL, NaCl 5.0 g/mL, pH 7.5) at 30 °C, 200 rpm for 24 h, unless specifically described.

**Table 2:** Strains and plasmids used in this study.

Strain or Plasmid	Description	Source or Reference
Strains		
*Acidovorax avenae* subsp. *avenae*		
RS-1	The pathogen of bacterial brown stripe of rice, isolated from the diseased rice from Zhejiang province in China. Wild type strain in this study	Lab collection
Δ*vgrG-*2	Km^R^; RS-1 in-frame deletion mutation defective in vgrG-2	This study
Δ*vgrG*-2(vgrG-2)	Km^R^; Chl^R^; complementary strain of Δ*vgrG-*2 complemented with pRADK-vgrG2	This study
Δ*vgrG*-2(pRADK)	Km^R^; Chl^R^; mock strain of Δ*vgrG-*2 with empty pRADK	This study
*Burkholderia seminalis* R456	Isolated from rice rhizosphere from Zhejiang province in China. Biocontrol bacterium used in this study	Lab collection
*Escherichia coli* S17-1 λ pir	λ Lysogenic S17-1 derivative producing π protein for replication of plasmids carrying R6K*ori*; *recAprohsdR*RP4-2-Tc::Mu-Km::Tn7 λ^−^pir	Liu *et al.* (2012) [47]
Plasmids		
pJP5603	Suicide vector; R6Kori, Km^R^	Liu *et al.* (2012) [47]
pJP-G	Km^R^; pJP5603 containing the 440 bp DNA fragment of gene *vgrG*-2 from Strain RS-1; used to create mutant Δ*vgrG-2*	This study
pRADK	Chl^R^; broad host expression vector	Liu *et al.* (2012) [47]
pRADK-vgrG2	Chl^R^; pRADK plasmid containing the *vgrG*-2 gene and upstream fragment from strain RS-1, utilize to complement	This study

Km^R^, Chl^R^: Kanamycin- and Chloromycetin-resistant, respectively.

### 4.2. Bacterial Growth in High Salt, Low Temperature and Oxidative Stress Conditions

In order to find the appropriate stress repression point for *Aaa* RS-1, the growth of the bacteria was firstly determined in different stress conditions of different concentrations. Bacterial adaptation to different environments was determined by examining cell growth in high salt, low temperature, and oxidative stress conditions. The 96-well microplates (Corning-Costar Corp., Corning, NY, USA) were used for this purpose. For salt stress treatment, each well in the 96-well microplates was inoculated with 200 µL of bacterial suspension (OD_600_ = 0.1) with NaCl concentrations of 1.0% (Optimum concentration, served as the control), 2.0%, 3.0%, 4.0%, 5.0%, 6.0%, 7.0%, 8.0%, and 9.0%. For low temperature treatment, bacterial growth was determined after incubating 200 µL of bacterial suspension (OD_600_ = 0.1) at 15 and 30 °C (Optimum temperature, served as the control) for 0, 3, 6, 12 and 24 h, respectively. For H_2_O_2_ treatment, bacterial suspension was incubated with 0.0 (the control), 8.0, 16.0, and 32.0 mM of H_2_O_2_ at 30 °C for 24 h. The effect of paraquat on bacterial growth was determined by incubating the bacterial suspension (OD_600_ = 0.3) with 0.0 (the control), 10.0, 50.0, and 100.0 μM of paraquat, at 30 °C for 0, 8, 16, and 24 h, respectively. The NB without bacteria was used as the negative control for this experiment. Finally, the OD_600_ of each plate was determined and bacterial growth was evaluated based on the OD_600_ of six biological replicates.

### 4.3. Bacterial Growth in Co-Culture and in Vivo Planta Conditions

The effect of co-culture on T6SS gene expression was examined by incubating *Aaa* RS-1 either alone or in combination with *B. seminalis* R456 according to Ruiz *et al.* (2009) [48]. Briefly, both bacterial strains were incubated in 40 mL of NB for overnight at 30 °C. In order to allow the interchange of secreted molecules present in the supernatants, the content of each tube was drawn in a sterile syringe and a sterile filter of 32 mm diameter and 0.45 μm pore size (Pall–Newquay) was connected to each syringe. Both syringe–filter sets, each one containing one bacterial strain, were interconnected by means of single and sterile fused-silica tubing, and the media of both syringes were manually mixed every 2 h. Batch cultures for each strain were performed at the same time and the whole procedure was independently repeated at least twice.

The bacterial growth during *in vivo* rice infection was determined by collecting bacteria from diseased rice leaves directly as described in our previous study [32]. Briefly, bacterial strain was inoculated and recovered as follows. Leaves of four-week-old plants were infiltrated with sterilized syringe filled with 10 mL (~10^8^ CFU/mL) of bacterial suspension. Six days after the inoculation, infected leaves were collected and decontaminated with alcohol. Leaves were cut into pieces with a sterile razor blade and maintained for 1 h in sterile glass plates containing 20 mL of distilled water. The incubation during this period of time allowed the bacteria to detach from the leaf tissues. The leaves were separated from the suspension and the bacterial cells were collected by centrifugation at 5000 RCF for 20 min. The bacterial cell pellets were then washed with phosphate buffer saline (PBS) and with water and then used for RNA extraction.

### 4.4. Construction of vgrG-2 Mutant and Complementation

The effect of gene mutation on bacterial growth and T6SS gene expression was determined by constructing one *vgrG* mutant of *Aaa* RS-1. In-frame deletion of *vgrG*-2 gene and complementation were performed as described of Liu *et al.* (2012) [47] by suicide plasmid pJP5603 through homologous recombination on the background of wild-type strain RS-1. For construction of the *vgrG*-2 deletion strain, 205 bp internal DNA fragment of *vgrG*-2 was PCR amplified with primers designed according to *Aaa* RS-1 genome. The PCR product was cloned into pMD19-T vector (TaKaRa, Dalian, China), verified by sequencing and digestion with BamHI and EcoRI, and then ligated into the suicide vector pJP5603 to get pJp-G. For transfer of plasmid, the *E. coli* S17-1 λ pir has been employed and the resulting plasmid was then introduced into *Aaa* RS-1 via electroporation. This *vgrG*-2 gene may be targeted during transcription because of the high similarity between the internal fragments harbored by suicide plasmid and genomic DNA. The homologous recombination mutants were obtained after a single integrative recombination event, which disconnected essential protein domains, resulting in truncated and non-functional VgrG-2 protein. Mutant checking of the *vgrG*-2 among eight *vgrG*s of high sequence similarity were further confirmed by examining the expression of either side of the part knocked out using qPCR. In order to complement the Δ*vgrG*-2 strain, *vgrG*-2 open reading frame was amplified from the wide strain, along with 300 bp upstream of its start codon so that it included its native promoter. The 2652 bp PCR product was cloned into pGEM-T Easy vector, verified by sequencing, and then cloned into pRADK. The complementation vector was introduced into mutant cells by filter mating and selected by Chl + Km resistance. In addition, mock strain was constructed by introducing empty pRADK into mutant cells. The primers were listed in Table 3.

**Table 3:** Primers of T6SS genes used for quantitative real-time PCR (qPCR) of *Acidovorax avenae* subsp. *avenae* RS-1 in this study.

T6SS Gene	Primer Sequence(5′→3′)	Target PCR Product of Function	Amplication Size (bp)
*clpB*	F-GCAGGGCGAGAAGGACAAG	ATP-dependent chaperone ClpB	159
	R-GCCGAGGAACAGGAACGAG		
*impA*	F-CTTGAACCTGCGGCGGACAC	Type VI secretion-associated protein, ImpA family	129
	R-GCTCGGCGGGAATCACCAT		
*impB*	F-ATCTCCCTCATCCTGCTCA	Hypothetical protein Aave_2851	152
	R-TCAGATGCGTCCCATCAG		
*duf877*	F-GCACCACCTGGTCCACAACA	Type VI secretion protein EvpB	163
	R-CGAACTGGCCGTATTCCTCT		
*impE*	F-TGATCGGCTCGCTGTTCG	Guanosine monophosphate reductase	120
	R-TGCTTGTACTCGCCCTTGTT		
*impF*	F-TGGACTGGAAGGACGTGGAA	Type VI secretion system lysozyme-like protein	126
	R-AGGGTGTTGTGGTGGTTGAA		
*impH*	F-TGGAACTTCGGCCTCTATGG	Type VI secretion protein	121
	R-TGGTGGAAGATGTCCGAGAA		
*pppA*	F-AGATCACGCGGGACCATT	Protein serine/threonine phosphatase	214
	R-TTCCTCGTCGTCGAGCAT		
*lip*	F-GCAGTGCGGATGTCCGTACCTT	Type VI secretion lipoprotein	174
	R-TCCTTGCCCACCGTGATGCT		
*impJ*	F-TCCAGGATGCCAACGACA	Type VI secretion protein, VC_A0114 family	181
	R-GACCACGGTGGGAATGAA		
*dotU*	F-CCAGCATTACCTGCTCGAAT	DotU family type IV/VI secretion system protein	196
	R-CCAGGTCTCGTTGTGCAGT		
*icmF*	F-ACCGTGGGCAGCAATCTCA	Type VI secretion protein IcmF	112
	R-GCGAAGTCATCGCTCGTCA		
*impM*	F-GCAATGGCGTCGTCCTCT	Adenylosuccinate synthase	192
	R-CGGTCGTGCCGATCTTCT		
*duf1305*	F-GCCACAAGTTCCTTTTGCA	Type VI secretion protein,VC_A0111 family	202
	R-AAGAACGGCACGAAATCC		
*vgrG-1*	F-ATCCGATGGAAAAGAAACTC	Rhs element Vgr protein Aave_0481	113
	R-AATAGATGCCCTCGTGCT		
*vgrG-2*	F-GCGTGCAATATGACGAGAGC	Rhs element Vgr protein Aave_0497	174
	R-CCGGCGGATAGAAGGGAATC		
*vgrG-3*	F-CGCACGATGCCTACGAGAT	Rhs element Vgr protein Aave_2047	121
	R-TTCGCCTTTGACGACGCT		
*vgrG-4*	F-CTGACGCAGAGCACGAAT	Rhs element Vgr protein Aave_2127	150
	R-CCGAAGCACCACATACCA		
*vgrG-5*	F-CATCAAGACCAAGTCCAGC	Rhs element Vgr protein Aave_2735	114
	R-CAGCCATAATTGCTCTGC		
*vgrG-6*	F-ATACTGCGTGCAATATGACG	Rhs element Vgr protein Aave_2840	185
	R-GATTTCTCGGGCGGATAG		
*vgrG-7*	F-CCGATGGAAAAGAAACTCAG	Rhs element Vgr protein Aave_3347	111
	R-AATAGATGCCCTCGTGCT		
*vgrG-8*	F-TCCTTCCAGAAGTTCAGCC	Rhs element Vgr protein Aave_0241	144
	R-GGTATTCGTCGGTCCAGATT		
vgrG-2s	F-TACCCGCCCGAGAAGT	Forepart fragment of the knockout fragment in *vgrG*-2	169
	R-CCGGCCATTCGTAGATC		
vgrG-2b	F-ACGGGTGTCTTCAAGATGG	Tail fragment of the knockout fragment in *vgrG*-2	197
	R-TGAGGGTGATGCTGGTTT		
CvgrG-2	F-ACACCACTTCGACGAGGTGCTG	*vgrG*-2 open reading frame with its promoter region	2652
	R-TCAGTTCAGGTGGATGTCTTCGC		
16s RNA	F-TTGCGGTCCCCTGCTTTCAT	Reference gene used for qPCR in this study	120
	R-CGGTAACAGGTCTTCGGATGCT		

### 4.5. RNA Extraction and Gene Expression Analysis Using Quantitative Real-Time PCR

This experiment was conducted to assess the expression pattern of T6SS gene in *Aaa* RS-1 cells subjected to different stress treatments including: incubation in NB with 2.0% of NaCl for high salt stress, incubation at 15 °C for low temperature stress, incubation in NB with 8.0 mM of H_2_O_2_ and incubation in NB with 50.0 μM of paraquat for oxidative stresses, co-culture with *B. seminalis* R456 and finally inoculated and recovered from the host plant. Bacteria incubated in NB with 1.0% of NaCl at 30 °C were used as the control. All the *in vitro* treatments were cultured for 24 h. Total RNAs from each sample was extracted by using high pure RNA isolation kit (Roche, Hangzhou, China) according to the manufactory instructions. RNA was treated with DNase I and reverse-transcribed into cDNA using a Prime Script™ RT reagent Kit with gDNA Eraser (TaKaRa, Dalian, China). The resulting cDNAs were used as the template for expression detection analysis of the T6SS gene with qPCR using a SYBR^®^ Premix Ex Taq™ (TaKaRa, Dalian, China) following the instruction of kit manual on an ABI Prism 7500 sequence detection system (Applied Biosystems, Foster City, CA, USA). The gene-specific primer sequences are shown in Table 3. The average threshold cycle (*C*_t_) was used to determine the fold change of gene expression. In addition, 16S rRNA gene was used as an internal control. The 2^−ΔΔ*C*t^ method was used for relative quantification [49]. Each result represents the average of three independent determinations. This experiment was repeated twice.

### 4.6. Principle Component Analysis of Gene Expression

The PCA method was used for the data of T6SS gene expression under different conditions. The RE was subjected to PCA after standardizing to unit variance. Resulting factor scores of the PC1 and PC2 were tested in two-way analysis of variances (ANOVA). Data analyses were carried out using the SPSS 16.0 software (SPSS, Michigan Avenue, Chicago, IL, USA). The figures were produced by using the SigmaPlot 10.0 software (SYSTAT Software, Inc., San Jose, CA, USA).

### 4.7. Statistics Analysis

The software STATGRAPHICS Plus, version 4.0 (Copyright Manugistics Inc., Rockville, MD, USA) was used to perform the statistical analysis. Levels of significance (*p* < 0.05) of main treatments and their interactions were calculated by analysis of variance after testing for normality and variance homogeneity.
